# Commentary on LRAs targeting NF‐κB with epigenetic and mutational impacts on HIV latency

**DOI:** 10.1002/imo2.31

**Published:** 2024-09-25

**Authors:** Shaoming Chen

**Affiliations:** ^1^ Rheast LLC Houston Texas USA

## Abstract

Human immunodeficiency virus (HIV) latency is controlled by factors like nuclear factor kappa B (NF‐κB), which binds to the long terminal repeat of the HIV genome to start viral gene expression. The primary cellular form of NF‐κB is a heterodimer comprising the DNA‐binding subunit p50 and the transactivator p65. Phosphorylation of IkappaB kinase (IκB) is driven by the IκB kinase complex, whose core is formed by the NF‐κB essential modulator. However, epigenetic changes like DNA methylation and histone modifications can suppress this activation. Recent studies show that HIV reservoirs are diverse, with complex interactions between viral and host factors affecting latency‐reversing agent (LRA) effectiveness. Mutations in the NF‐κB binding sites, converting them to GA‐binding protein sites, complicate latency reversal by altering responses to LRAs.

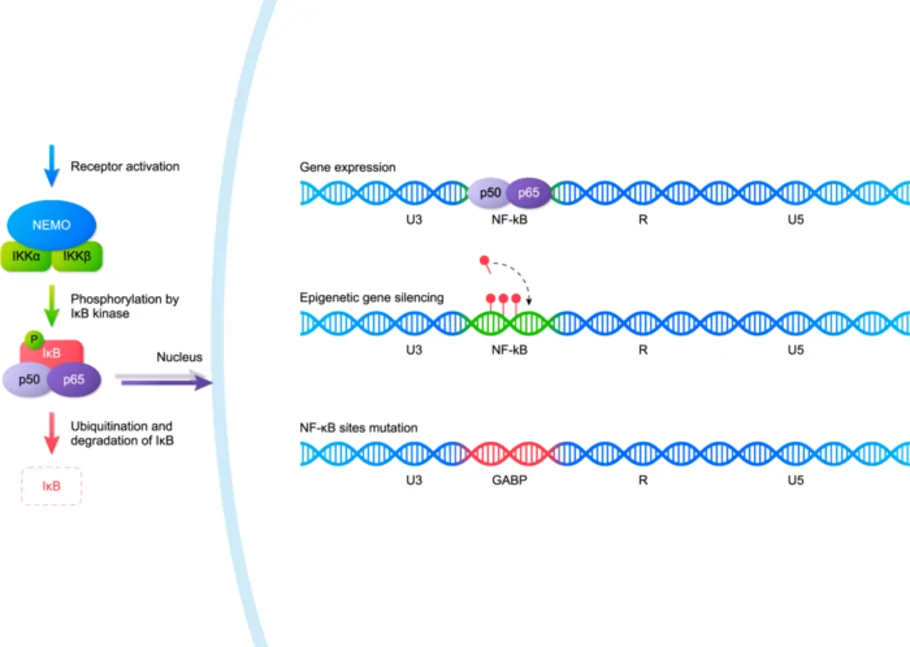

Acquired immunodeficiency syndrome is caused by the human immunodeficiency virus (HIV), which targets immune system cells and replicates using their machinery. Some HIV‐infected cells enter a latent state, not producing new virus, forming a reservoir where HIV can hide for years and evade therapy. These cells can reactivate anytime to produce more viruses. A major challenge in curing HIV is its ability to remain latent in immune cells like cluster of differentiation 4 (CD4) cells, making antiretroviral therapy (ART) ineffective during latency. Researchers are exploring latency‐reversing agents (LRAs) to reactivate latent HIV in CD4 cells, allowing ART and the immune system to fight the virus, but LRAs still have not been approved by the Food and Drug Administration [1]. Hence, further investigation is needed to determine the applicability of this method.

HIV latency is a complex phenomenon where the virus remains latent within cells, evading the immune system and antiretroviral drugs. The promoter‐proximal (enhancer) region of the HIV‐1 long terminal repeat (LTR) contains two adjacent nuclear factor kappa B (NF‐κB) binding sites that play a central role in mediating inducible HIV‐1 gene expression [2]. NF‐κB is a transcription factor regulating immune responses and inflammation by controlling gene activation. In HIV‐1, NF‐κB binding sites in the LTR's promoter‐proximal region allow these transcription factors to bind and initiate HIV‐1 gene transcription upon activation. This leads to viral RNA production and new virus particles. These NF‐κB binding sites enable the virus to control replication based on the host environment, staying latent under unfavorable conditions and activating when conditions improve. This presents a potential therapeutic target: manipulating NF‐κB activation could control HIV‐1 gene expression, offering strategies to suppress viral replication or reactivate latent virus for elimination [3].

## 1 THE IMPACT OF EPIGENETIC

Clinical trials of LRAs within the “shock and kill” strategy have produced unconvincing results. Recent studies reveal varied responses of infected cells to LRAs, highlighting their limited effectiveness and the numerous factors contributing to reservoir heterogeneity. These factors include virus genetic background, cell model, cell type, silencing mechanisms, tissue reservoirs, integration sites, patient‐specific factors, and gender. Additionally, studies show conflicting observations on the impact of LRAs on natural killer and cytotoxic T‐cell activity, indicating either an immunosuppressive effect or reduced influence on cells sensing HIV‐1 reactivation [4].

Several studies indicate that AZD5582 can reactivate latent HIV and simian immunodeficiency virus, but its effectiveness is only 42% [5, 6]. Notably, the novel small‐molecule IAP inhibitor AZD5582 has been used for the treatment of cancer and reportedly causes cIAP1 degradation, and thus induces apoptosis in the MDA‐MB‐231 breast cancer cell line at subnanomolar concentrations in vitro. [7] Other LRAs like disulfiram [8], bryostatin [9], ingenol [10], and prostratin [11] are also noted for reactivating latent HIV. These LRAs are used in cancer treatment: disulfiram inhibits prostate cancer growth [12], bryostatin shows strong antitumor activity [13], ingenol compounds are effective against various cancer cell lines [14], and prostratin inhibits SIK3 displaying potential anticancer effects [15].

Epigenetic constraints play a crucial role in suppressing latent HIV transcription, beyond the involvement of transcription factors like NF‐κB. These modifications, such as DNA methylation and histone changes, alter gene expression without changing the DNA sequence, creating a repressive environment that keeps the virus inactive. Even when NF‐κB is activated, it may not overcome these epigenetic barriers, meaning latent HIV remains unresponsive to LRAs that activate NF‐κB. This “brake” on the transcriptional machinery prevents the activation of latent viruses, showing that NF‐κB activation alone is insufficient to trigger latent HIV.

## 2 THE IMPACT OF MUTATION

HIV‐1 is classified into four groups (M, N, O, and P) with Group M being the most prevalent and subdivided into nine subtypes (A–D, F–H, J, and K), circulating recombinant forms (CRFs), and unique recombinant forms. Studies have shown that different HIV‐1 strains impact transmission, replication, pathogenesis, diagnosis, and therapy response. Subtype A is less pathogenic and replicates slower than subtype C, but has a higher heterosexual transmission rate compared to subtype D and CRF01_AE compared to subtype B. Subtype D is linked to faster disease progression than A, C, and CRFs. Genetic variability within and between clades affects HIV‐1's biological properties, particularly the LTR's role in replication and expression. For instance, a mutation converting an NF‐κB site to a GA‐binding protein (GABP) binding site increases CRF01_AE's replication and transmission over subtype B [16].

GABP is a transcription factor that plays a key role in regulating gene expression. It is a member of the E26 transformation‐specific (ETS) family of transcription factors, which are characterized by their ability to bind to specific DNA sequences known as ETS motifs. GABP is a heterotetramer composed of GABPα and GABPβ subunits, and it is involved in various cellular processes. Research identified a single‐nucleotide deletion in the upstream NF‐κB site of the HIV‐1 LTR promoter's tandem enhancer motif, selected during long‐term culture of a Tat‐defective HIV‐1 mutant. EMSAs showed a loss of NF‐κB binding and basal promoter activity. Despite this, the mutation improved virus replication, with a new binding activity specific to the mutant LTR emerging in EMSAs from unstimulated cells. This complex did not react with NF‐κB‐specific antibodies but was identified as GABP through supershift assays. The deletion reduced NF‐κB binding by eightfold while increasing GABP affinity by fourteenfold. This adaptation is not unique, as subtype E HIV‐1 LTR sequences align with the new GABP/NF‐κB enhancer configuration. All 18 subtype E isolates tested have this GABP site, with NF‐κB binding abolished but without loss of promoter function [17].

If NF‐κB undergoes a mutation that transforms its function or structure to resemble that of GABP, the efficacy of LRAs specifically targeting NF‐κB would likely be compromised. This is because LRAs are designed to interact with the distinct molecular features and regulatory pathways associated with NF‐κB. A mutation that significantly alters NF‐κB's structure or functional properties could prevent these agents from binding effectively or modulating NF‐κB activity as intended.

## 3 FUTURE DIRECTIONS

**Figure 1:**
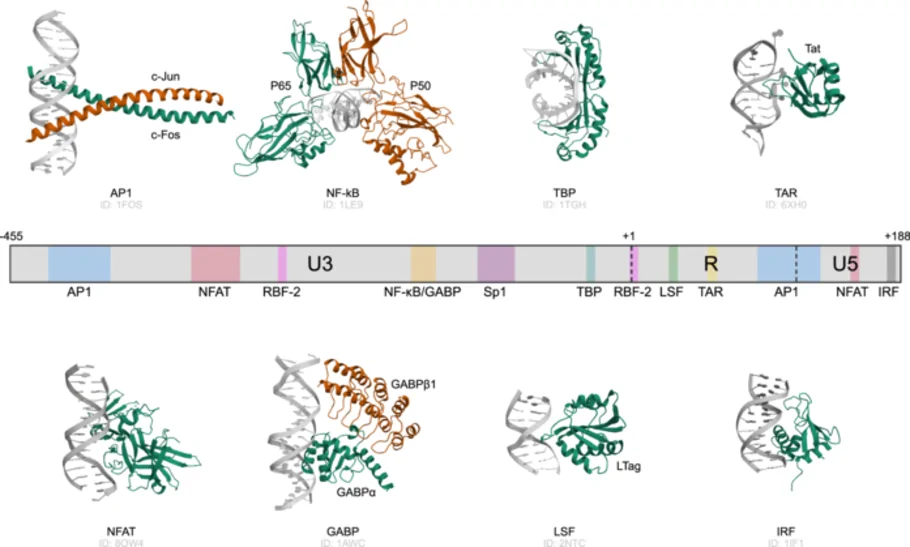
HIV‐1 5′LTR promoter. A schematic of the HIV‐1 5′‐long terminal repeat (LTR) promoter highlights its functional domains, which include multiple *cis*‐regulatory elements that interact with various host cellular factors. The transcription start site is situated at the boundary between the U3 and R regions. The 5′LTR region contains several important transcription factor binding sites, including Activator Protein 1 (AP1), GA‐binding protein (GABP), Interferon Regulatory Factor (IRF), Late SV40 Factor (LSF), Nuclear Factor kappa‐light‐chain‐enhancer of activated B cells (NF‐κB), Nuclear Factor of Activated T‐cells (NFAT), RNA Polymerase II Binding Factor 2 (RBF‐2), Specificity Protein 1 (Sp1), TATA‐box binding protein (TBP), Trans‐Activation Response Element (TAR).

Besides NF‐κB and GABP, there are other important transcription factors and elements that play roles in gene regulation, including Activator Protein 1 (AP1), Specificity Protein 1 (Sp1), Octamer‐binding Transcription Factor 1 (Oct1), and Trans‐Activation Response Element (TAR), as shown in Figure 1. AP1 is a transcription factor made up of proteins from the Jun, Fos, Activating Transcription Factor, and Jun Dimerization Protein families, which regulates genes in response to various stimuli, such as growth factors, stress, and cytokines, influencing cell proliferation, differentiation, and apoptosis. Sp1 binds to guanine‐cytosine rich (GC‐rich) regions in gene promoters and enhancers, modulating the expression of genes involved in cell growth, differentiation, and responses to environmental signals. Oct1 binds to the octamer motif found in many gene regulatory regions, playing a role in regulating gene expression across different tissues and developmental stages, and is positioned close to the binding site of RNA Polymerase II Binding Factor 2. The TAR is a sequence in the HIV‐1 genome that interacts with the viral Tat protein, significantly enhancing the transcription of viral genes [16, 18].

For example, Tat is a protein ranging from 86 to 101 amino acids in length, depending on the subtype. It significantly enhances HIV dsDNA transcription. Initially, in the absence of Tat, only a few RNA transcripts are produced, which in turn allows for Tat production. Once present, Tat binds to cellular factors and facilitates their phosphorylation, leading to increased transcription of all HIV genes and creating a positive feedback loop [19]. The Tat/Cyclin T1/CDK9 complex plays a central role in HIV‐1 trans‐activation, with its affinity for TAR regulated by Tat acetylation through histone acetyl transferases. Specific mutations in TAR RNA can significantly impair HIV‐1 trans‐activation, translation, and viral production, underscoring its essential role [20].

The future goal is to combine multiple targets for more comprehensive latency reversal strategies, but targeting these sites to reactivate latent HIV presents challenges. Unlike conventional agents, Tat proteins are not small molecular compounds that can be directly administered, and require specialized delivery systems to effectively reach their targets within the cell. Furthermore, these strategies necessitate rigorous clinical validation to confirm their efficacy and safety in reactivating latent HIV reservoirs.

## AUTHOR CONTRIBUTIONS

**Shaoming Chen**: Writing—review and editing; writing—original draft.

## CONFLICT OF INTEREST STATEMENT

The author declares potential conflicts of interest regarding the review of this research. Given that the study challenges the efficacy of LRAs and proposes alternative methodologies, the author recommend that individuals involved in LRAs research refrain from participating in the peer‐review process to avoid conflict of interest.

## ETHICS STATEMENT

No animals or humans were involved in this study.

## Data Availability

The public 3D models were obtained from the RCSB PDB (https://www.rcsb.org) with the following IDs: 1FOS, 1LE9, 1TGH, 6XH0, 8OW4, 1AWC, 2NTC, and 1IF1. Supplementary materials (graphical abstract, slides, videos, Chinese translated version and update materials) may be found in the online DOI or iMeta Science, http://www.imeta.science/imetaomics/.
